# *Moringa oleifera*: A Review of the Pharmacology, Chemical Constituents, and Application for Dental Health

**DOI:** 10.3390/ph17010142

**Published:** 2024-01-22

**Authors:** Meiny Faudah Amin, Taufiq Ariwibowo, Salsabila Aqila Putri, Dikdik Kurnia

**Affiliations:** 1 Department Conservative Dentistry, Faculty of Dentistry, Universitas Trisakt, Jakarta Barat 11440, Indonesia; taufiq@trisakti.ac.id; 2 Department of Chemistry, Faculty of Mathematics and Natural Sciences, Universitas Padjadjaran, Sumedang 45363, Indonesia; salsabila21021@mail.unpad.ac.id (S.A.P.); dikdik.kurnia@unpad.ac.id (D.K.)

**Keywords:** *Moringa oleifera*, pharmacological use, phytochemical constituent

## Abstract

*Moringa oleifera* L., commonly known as Kelor in Indonesia and miracle tree in English, has a rich history of utilization for medicinal, nutritional, and water treatment purposes dating back to ancient times. The plant is renowned for its abundance of vitamins, minerals, and various chemical constituents, making it a valuable resource. Among its notable pharmacological properties are its effectiveness as an anti-diabetic, anti-diarrheal, anti-helmintic, anti-leishmanial, anti-fungal, anti-bacterial, anti-allergic, anti-cancer, anti-inflammatory, and anti-oxidant agent. In this comprehensive review, we delve into the extensive pharmacological applications and phytochemical constituents of *M. oleifera* and its application in dental health.

## 1. Introduction

*Moringa oleifera* L. comes from the Moringaeceae family and is commonly known as Kelor in Indonesia, Sahajan in India, and Horseradish tree or Drumstick tree in English. It is also described as a miracle tree due to its nutritional value, diverse functions, and medicinal properties. *M. oleifera* can grow up to 12 m in tropical and subtropical environments. Although it is native to South Asia, the cultivation itself has already spread to the Middle East, Africa, Asia, and other areas. Traditionally, *M. oleifera* has been used in medicine, skincare, breastmilk production, and even food. Almost all parts of *M. oleifera* can be useful. Nowadays, *M. oleifera* is also used for water purification, animal feed, as a bio-stimulant, bio-pesticide, and biomass as biodiesel production in industrial and agricultural processes [1,2,3,4,5,6]. Table 1 describes the traditional usage of *M. oleifera*.

As a food and stimulant, *M. oleifera* is known to have an abundance of nutritional value and is comparatively easy to cultivate because of its rapid growth and good adaptability to climate change. Thus, in poor countries, *M. oleifera* is used as a source of proteins, calories, minerals, and vitamins. It has been reported that dry leaves of *M. oleifera* contain more calories, protein, carbohydrate, fiber, vitamin B, calcium, magnesium, phosphorus, potassium, copper, and iron than fresh leaves. Meanwhile, fresh leaves contain more vitamin C and E. Between the leaf, seed, and pod of *M. oleifera*, proteins, vitamin E, and magnesium have been found to be more abundant in the seed [7,8,9].

Due to many its traditional usages, research has been conducted to prove its ability as medicine. It is reported to have pharmacological properties such as anti-diabetic, anti-diarrheal, anti-helmintic, anti-leishmanial, anti-fungi, anti-bacterial, anti-allergic, anti-cancer, anti-inflammatory, and anti-oxidant. Hastuty and Nitia reported the efficiency of *M. oleifera* leaf extract to raise hemoglobin levels in young girls. They showed a value of 10.83 g/dL before treatment, while after treatment with *M. oleifera,* the hemoglobin levels increased to 12.72 g/dL [10]. Yuliastuti and Kurnia also reported the effect of *M. oleifera* on hemoglobin levels in anemic pregnant women. The result showed a significant difference, where before treatment, the respondents showed hemoglobin levels about 10.2 g/dL, and after treatment these increased to 10.8 g/dL (*p* = 0.003 < 0.05) [11]. 

Dental health has been a concern for researchers to this day. The various infections that can occur in the teeth cause a decline in health. Dental infections occur due to the growth of various kinds of microbes in the dental and oral area. Among these are odontogenic infections and periradicular periodontitis that occur in the root canal system caused by anaerobic bacteria such as *Porphyromonas gingivalis*, *Enteroccus faecalis*, and *Candida albicans* [12,13,14]. *E. faecalis* is reported to infect root canals up to 30–80% [15]. In addition, based on polymerase chain reaction (PCR) analysis, the bacteria *Tannerella forysthia*, *Treponema denticola*, *Dialister pneumosintes*, and *Prevotella tannerae* were also reported to infect root canals with high prevalence [16]. These infections occur due to biofilm formation by microbes on the tooth area [17]. The use of antibiotics is a very useful treatment due to their effectiveness, low cost, and compatibility. However, resistance to antibiotic agents by microbes has been identified. Gram-negative bacteria have been reported to be resistant to beta-lactam antibiotics due to an enzyme that can open the ring in the beta-lactam structure, thus inactivating the action of the drug [18]. 

Therefore, natural product drug discovery research is of particular interest to researchers as a natural antibacterial agent. The main parameters in determining antibacterial activity are the inhibition zone value, minimum inhibition concentration (MIC) as a reference for the minimum concentration that can inhibit bacterial growth, and minimum bactericidal concentration (MBC) as a reference for the minimum concentration needed to kill a microorganism [19]. Based on the bioactivity and phytochemicals contained in *M. oleifera*, this plant has become one of the natural sources for dental disease treatment. *M. oleifera* seed extract was reported to provide MIC and antibiofilm values against *P. gingivalis* of 12.5 mg/mL and 6.25 mg/mL, respectively [20]. *M. oleifera* nanosuspension can inhibit *Aggregatibacteri actinomycetemcomitans*, *P. gingivalis*, *Prevotella intermedia*, *Fusobacterium nucleatum* with an MIC and MBC of 25% and 12.5%, respectively [21]. Based on this description, this review will explain the bioactivity contained in *M. oleifera* and describe the role of the plant for dental health and the chemical components contained therein.

## 2. Phytochemical Constituent

The pharmacological effects of *M. oleifera* are influenced by its phytochemical components. Previous studies have reported that there are several groups of compounds that are unique to each part of *M. oleifera*. The flowers are known to contain flavonoids, alkaloids, sucrose, and amino acids such as kaempferitrin, isoquercitrin, and rhamnetin. Furthermore, the stem contains alkaloid compounds such as moringinine and moringin, octacosanoic acid, β-sitosterol, and 4-hydroxymellein. The seed contains high contents of 4-(α-l-rhamnosyloxy) phenylacetonitrile, benzylglucosinolate, 4-(α-l-rhamnosyloxy) benzylisothiocyanate, *O*-ethyl-4-(α-l-rhamnosyloxy) benzyl, and 4-(α-l-rhamnopyranosyloxy)-benzylglucosinolate carbamate, while the fruit contains cytokines. In addition, the whole pods were specific for *O*-[2′-hydroxy-3′-(2′′-heptenyloxy)]-propyl undecanoate, methyl-p-hydroxybenzoate, thiocarbamates, isothiocyanate, nitrile, and *O*-ethyl-4-[(α-l-rhamnosyloxy)-benzyl] carbamate [22]. The seeds of *M. oleifera* contain total flavonoids 144.07 mg/kg, total polyphenols 145.16 mg/100 g, and proanthocyanidines 140.49 mg/kg. In addition, the oil of *M. oleifera* contains 18.24 mg rutin equivalent/g (total flavonoids), 37.94 mg ascorbic acid equivalent/g (total antioxidant capacity), and 40.17 mg GA equivalent/g (total phenols). Based on this description, the following are some of the structures of phytochemical components contained in *M. oleifera* [23].

### 2.1. Phenolic

**Figure 1:**
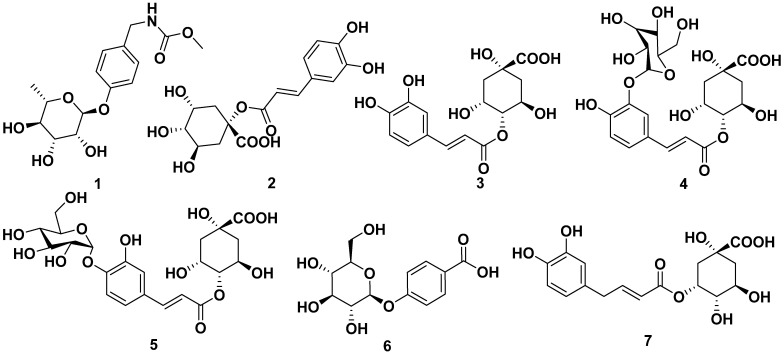

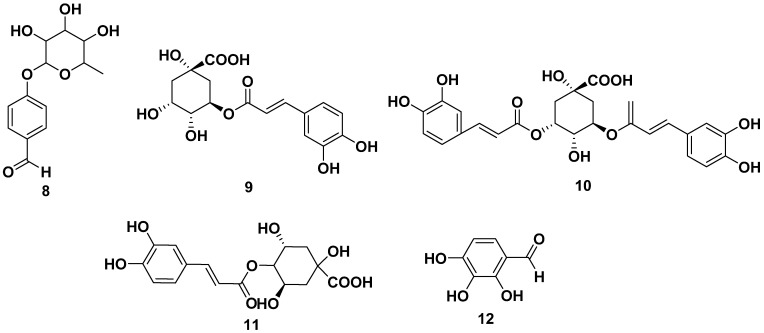
Phenolic compounds in ethanol and butanol extracts of seeds and leaves of *M. oleifera*.

Niazirin (**1**) was obtained through an ethanol and butanol extraction of the seeds and leaves of *M. oleifera*. It was reported to inhibit α-glucosidase inhibitor with an IC_50_ value of 382.2 µM. [24,25,26,27,28]. Caffeoylquinic acid (**2**), 4-*O*-caffeoylquinic acid (**3**), 4-*O*-(3′-*O*-α-D-glucopyranosyl)-caffeoylquinic acid (**4**), 4-*O*-(4′-*O*-α-D-glucopyranosyl)-caffeoylquinic acid (**5**), 4-*O*-β-D-glucopyranoside benzoic acid (**6**), 5-*O*-caffeoylquinic acid (**7**), benzaldehyde 4-*O*-α-L-rhamnopyranoside (**8**), chlorogenic acid (**9**), methyl caffeoylquinate (**10**), methyl 4-caffeoylquinate (**11**), and 3,4-dihydroxybenzoic acid (**12**) could be obtained through an ethyl acetate and butanol extraction of *M. oleifera* leaves. The structures of the compounds are shown in Figure 1 [24,25,26,27,28].

**Figure 2:**
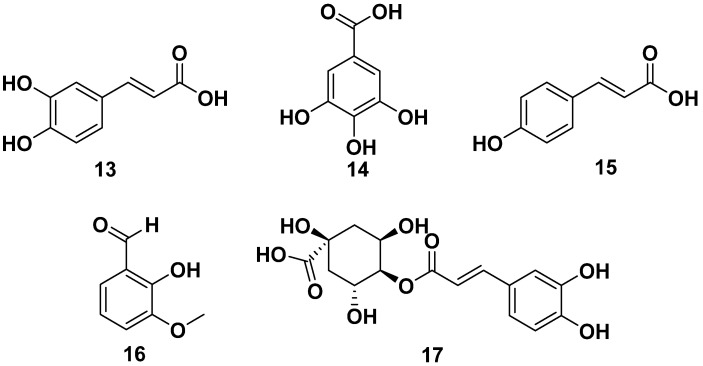
Phenolic compounds of other parts of *M. oleifera*.

Other phenolic compounds that could be isolated from various parts of *M. oleifera* were caffeic acid (**13**), gallic acid (**14**), *p*-coumaric (**15**), and vanillin (**16**). Cryptochlorogenic acid (**17**) also could be obtained from *M. oleifera*, and it was also reported to have anticancer activity against MCF-7 with an IC_50_ value of 20.8 M. The structures are shown in Figure 2 [28,29,30].

### 2.2. Glucosinolate

**Figure 3:**
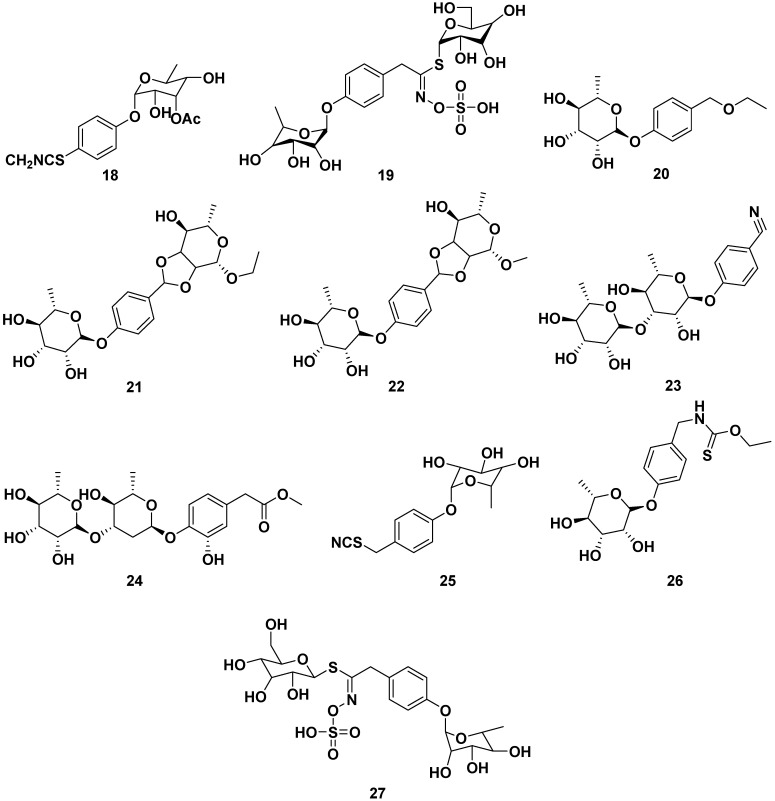
Glucosinolate compounds of seed ethanol extract of *M. oleifera*.

Glucosinolate compounds, as shown in Figure 3, could be isolated from the ethanol extracts of seeds of *M. oleifera*. These include the following compounds: 4-(3′-O-acetyl-α-L-rhamnosyloxy) benzyl isothiocyanate (**18**), 4-(α-L-rhamnopyranosyloxy)-benzylglucosinolate (**19**), 4-(α-L-rhamnosyl) benzyl ethyl ester (**20**), moringaside C (**21**), moringaside D (**22**), moringaside E (**23**), moringaside F (**24**), moringaside G (**25**), moringin (**26**), niazimicin (**27**), and glucomoringin (**28**). Furthermore, compound **23** exhibited α-glucosidase inhibitory activity with an IC_50_ value of 382.8 µM, meanwhile compound **26** had a reported an anti-adipogenic effect and an anticancer activity against HeLa cells with an IC_50_ value of 9.2 µg/mL. Meanwhile, compound **28** exhibited anti-allergic properties with IC_50_ values towards β-hexosaminidase and histamine releases of 10.43 and 27.22 µM, respectively. It is also reported to has antiviral properties against H1N1 with an IC_50_ value of 0.98 µg/mL [13,15,26,30,31,32,33,34,35,36,37].

**Figure 4:**
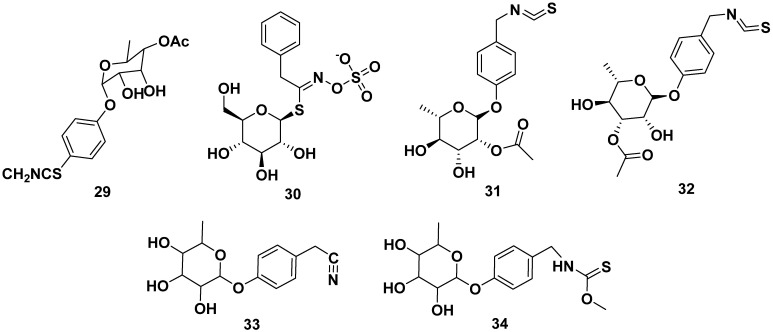
Glucosinolate compounds of other parts of *M. oleifera*.

Other glucosinolates found in *M. oleifera* were 4-(4′-O-acetyl-α-L-rhamnosyloxy) benzyl isothiocyanate (**29**), benzyl glucosinolate (**30**), 4-[(2′-*O*-acetyl-α-L-rhamnosyloxy) benzyl] isothiocyanate (**31**), 4-[(3′-*O*-acetyl-α-L-rhamnosyloxy) benzyl] isothiocyanate (**32**), niazinin (**33**), and niazinin B (**34**). Furthermore, compound **31** and **32** exhibited NO inhibitory activity with IC_50_ values of 1.67 and 2.66 µM, respectively, meanwhile compound **33** exhibited antileishmanial properties with an IC_50_ value of 5.25 mM. The structures are shown in Figure 4 [24,38,39,40,41].

### 2.3. Flavonoid

**Figure 5:**
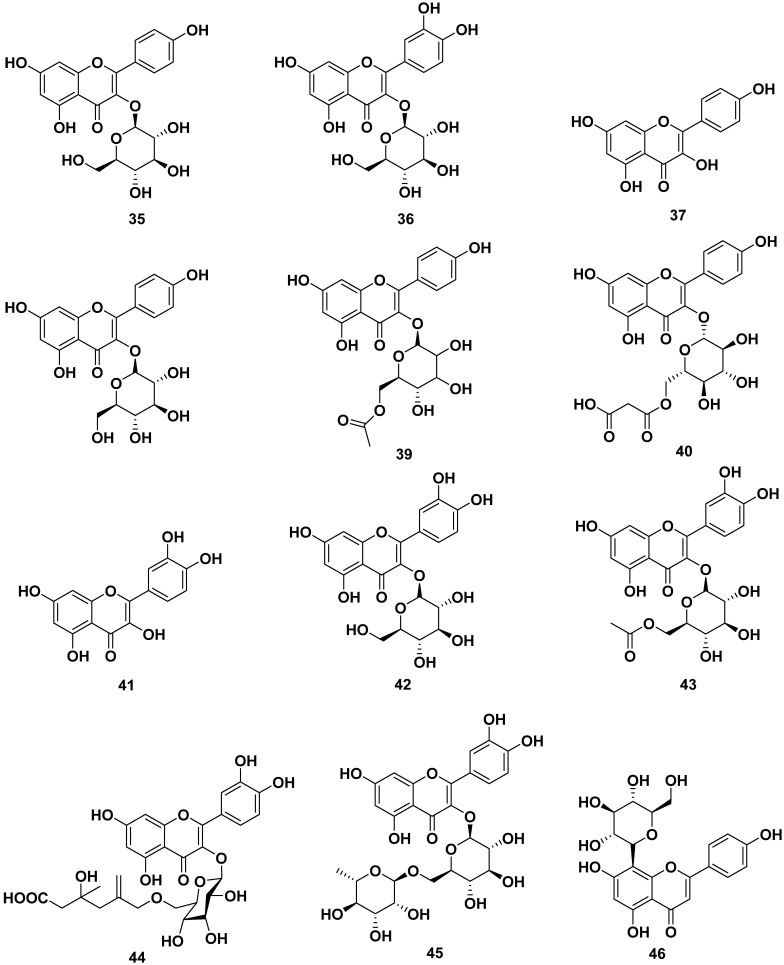

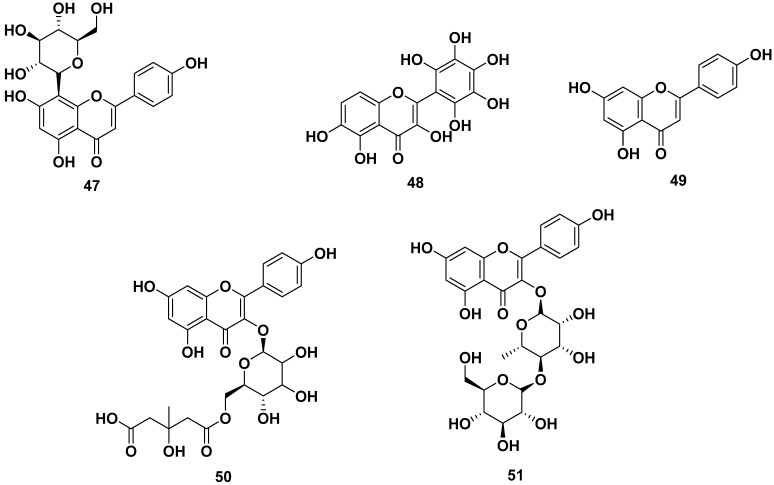
Flavonoid compounds of leaves, barks, and seeds of *M. oleifera*.

The leaves, barks, and seeds of *M. oleifera* contained various flavonoid compounds, as shown in Figure 5. The flavonoids reported were astragalin (**35**), isoquercitrin (**36**), kaempferol (**37**), kaempferol 3-*O*-glucoside (**38**), kaempferol acetyl glycoside (**39**), kaempferol-3-*O*-(6″-malonyl-glucoside) (**40**), quercetin (**41**), quercetin 3-*O*-β-D-glucopyranoside (**42**), quercetin-3-acetyl-glucoside (**43**), quercetin-3-*O*-(6″-malonyl-glucoside) (**44**), quercetin-3-*O*-β-D-(6″-*O*-3-hydroxy-3-methylglutaryl)-glucoside (**45**), rutin (**46**), vitexin (**47**), and 3,5,6-trihydroxy-2-(2,3,4,5,6-pentahydroxyphenyl)-4*H*chrome*n*-4-one (**48**). Those compounds could be obtained through extraction with methanol, ethanol, butanol, and ethyl acetate. Compounds **35**, **36**, and **46** were reported to inhibit CYP3A4 and CYP2D6, with IC_50_ values of 69.5 and 90 µM for compound **35**, whereas compounds **36** and **46** were reported to be CYP3AP inhibitors with IC_50_ values of 65.5 and 60 µM, respectively. 

Compounds **37**, **41**, and **48** were reported to have anti-allergic properties by inhibiting β-hexosaminidase and histamine release, with IC_50_ values of 29.39 and 46.94 µM, respectively, for compound **37**. Compound **41** exhibited IC_50_ values of 19.07 and 7.77 µM, respectively. Compound **48** showed IC_50_ values of 17.70 and 44.87 µM, respectively. Furthermore, compound **46** also inhibited α-glucosidase and pancreatic lipase with IC_50_ values of 40 and 35 µg/mL, respectively. Compound **47** also exhibited antiviral properties against virus H1N1 with an IC_50_ value of 3.42 µg/mL [13,24,25,26,27,33,39,42,43,44,45,46].

Apigenin (**49**), kaempferol-3-*O*-[methyl-(*S*)-3-hydroxy-3-methylglutaroyl(1→6)]-β-d-glucopyranoside (**50**), and multiflorin-B (**51**) also could be obtained from *M. oleifera* ethanol extracts [42,47,48].

### 2.4. Fatty Acid

**Figure 6:**
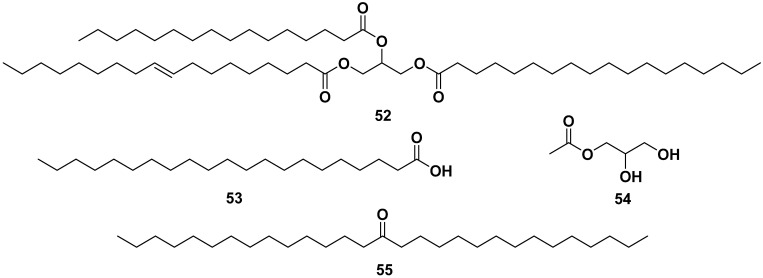

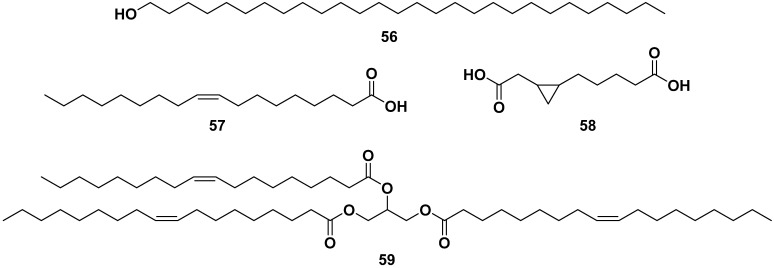
Fatty acid compounds of leaves, seeds, and flowers of ethanol, methanol, and ethyl acetate extracts of *M. oleifera*.

Figure 6 shows the structure of fatty acid compounds from the ethanol, methanol, and ethyl acetate extracts of leaves, seeds, and flowers of *M. oleifera*. The compounds are glycerol-1-(9-octadecanoate) (**52**), heneicosanoic acid (**53**), monoacetyl glycerol (**54**), monacosa*n*-15-one (**55**), octacosanol (**56**), oleic acid (**57**), 3,4-methyleneazelaic acid (**58**), and triolein acid (**59**). Compound **57** exhibited anti-allergic properties by inhibiting β-hexosaminidase and histamine release, with IC_50_ values of 53.76 and 56.05 µM, respectively [40,42,49,50,51,52,53].

### 2.5. Ester

**Figure 7:**
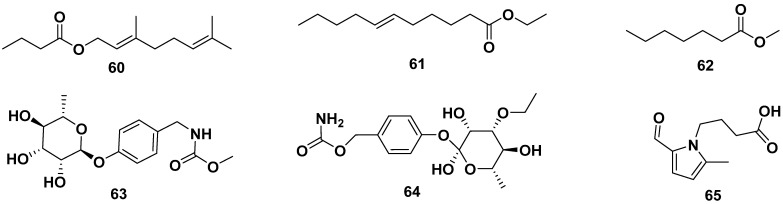
Ester compounds of leaves, flowers, and seeds of ethanol, methanol, and *n*-hexane extracts of *M. oleifera*.

Ethyl geranyl acetate (**60**), ethyl-*(E)*-undec-6-enoate (**61**), methyl heptanoate (**62**), methyl-4-(α-L-rhamnopyranosyloxy) benzyl carbamate (**63**), *O*-ethyl-4-(α-l-rhamnosyloxy)benzyl carbamate (**64**), and 2-formyl-5-methyl-1*H*-pyrrol-1-ylbutanoic acid (**65**) were ester groups isolated from leaves, flowers, and seeds of ethanol, methanol, and *n*-hexane *M. oleifera* extracts. The structures are shown in Figure 7. Compound **61** exhibited anti-allergic properties by inhibiting β-hexosaminidase and histamine release with IC_50_ values of 82.68 and 82.07 µM, respectively [42,49,50,53,54,55].

### 2.6. Alkaloid

**Figure 8:**
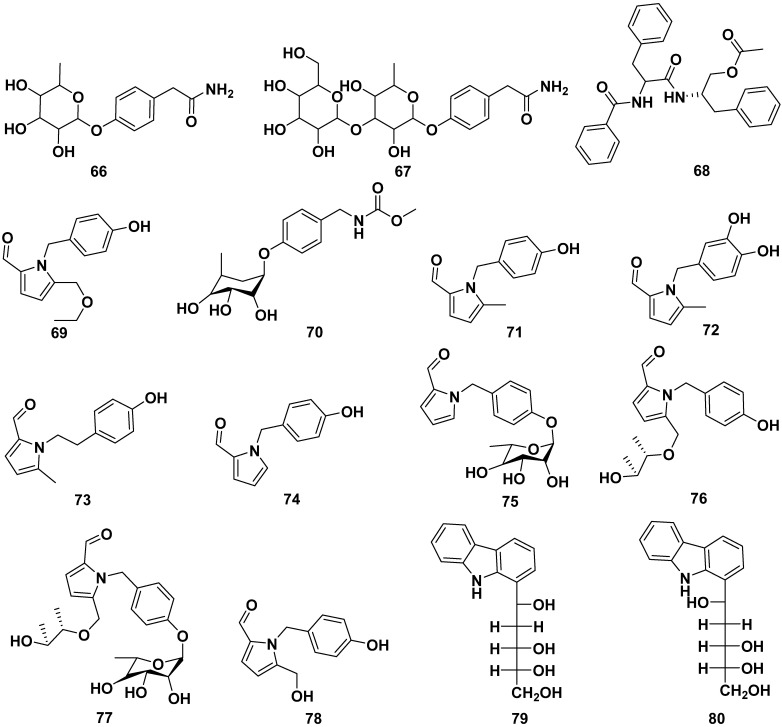
Alkaloid compounds of roots, seeds, and leaves of *M. oleifera*.

The roots, seeds, and leaves of *M. oleifera* contain alkaloids, as shown in Figure 8. Some of them could be obtained from butanol extraction. The alkaloids contained in *M. oleifera* include the following: marumoside A (**66**), marumoside B (**67**), aurantiamide acetate (**68**), hostine D (**69**), methyl 4-(α-L-rhamnopyranosyloxy) benzylcarbamate (**70**), pyrrolemorine A (**71**), pyrrolemorine B (**72**), pyrrolemorine C (**73**), pyrrolemorine D (**74**), pyrrolemorine E (**75**), pyrrolemorine F (**76**), pyrrolemorine G (**77**), pyrrolemarumine (**78**), tangutorid E (**79**), and tangutorid F (**80**). Compounds **71** and **75** demonstrated notable neuroprotective effects. At a concentration of 0.1 μM, they effectively mitigated PC12 cell damage caused by oxygen glucose deprivation and concurrently reduced the expression of NF-kB [42,47,56,57].

### 2.7. Sterol

**Figure 9:**
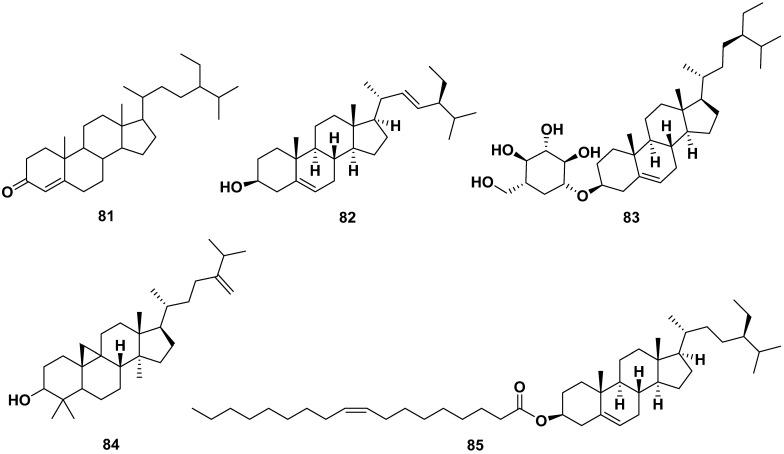
Sterol compounds of *M. oleifera*.

All parts of *M. oleifera* contain sterol compounds, as shown in Figure 9. The compounds could be isolated from methanol, ethanol, ethyl acetate, and acetone extracts. The following are sterols isolated from various parts of *M. oleifera*: β-sitosterone (**81**), stigmasterol (**82**), β-sitosterol-3-O-glucoside (**83**), β-sitosteryl oleate (**84**), and 24-methylene-9,19-cyclolanostan-3-ol (**85**). Compounds **82** and **83** exhibited anti-allergic properties by inhibiting β-hexosaminidase and histamine release with IC_50_ values of 75.92 and 38.27 µM, respectively, for compound **82**; meanwhile, compound **83** only inhibited β-hexosaminidase release with an IC_50_ value of 24.93 µM. Moreover, compound **82** exhibited anti-inflammatory characteristics by inhibiting caspase 1 and NF-kB. It also exhibited anti-adipogenic properties by reducing the S and G2/M phases, inhibiting ROS, and enhancing glucose uptake [13,31,37,58,59,60,61].

### 2.8. Terpene

**Figure 10:**
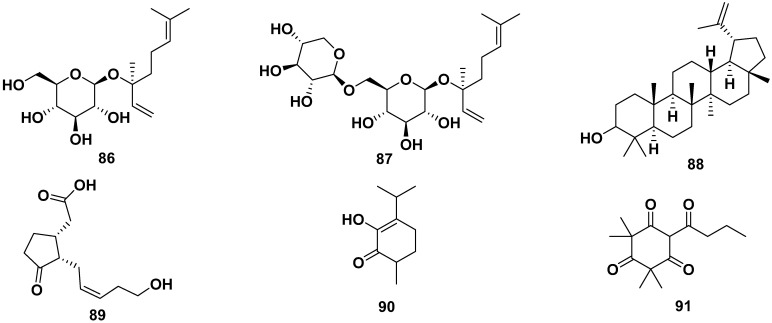
Terpene compounds of *M. oleifera*.

(*S*) Linalyl-β-D-glucoside (**86**), (S) linalyl-β-primeveroside (**87**), lupeol (**88**), tuberonic acid (**89**), γ-diosphenol (**90**), and 2,2,4,4-tetramethyl-6-(1-oxobutyl)-1,3,5-cyclohexanetrione (**91**) were terpenes isolated from *M. oleifera*. Compound **88** exhibited anti-adipogenic properties by reducing the S and G2/M phases, inhibiting ROS, and enhancing glucose uptake. The structure of the compounds is shown in Figure 10 [52,54,62].

### 2.9. Other Compounds

**Figure 11:**
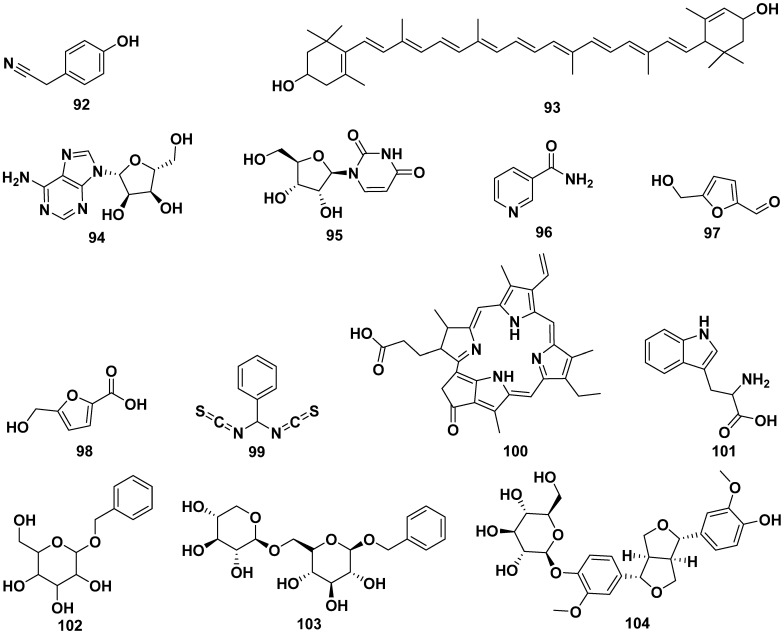

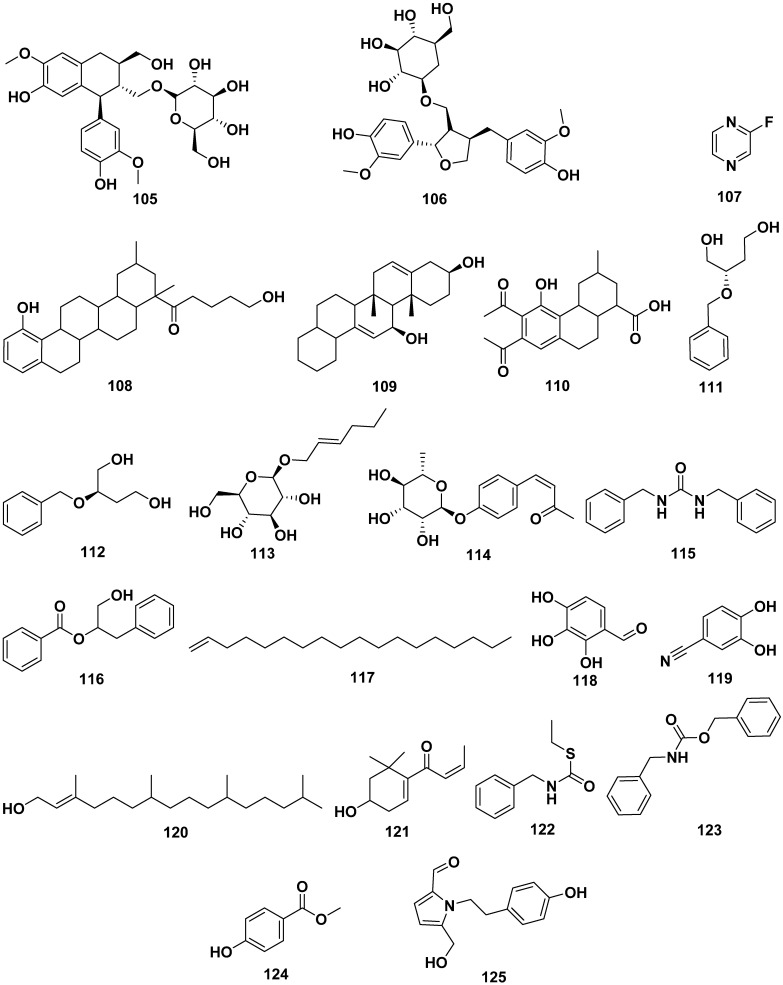
The structures of compounds of leaf extract of *M*. *oleifera*.

The leaf extract of *M. oleifera* contained 4-hydroxyphenylacetonitrile (**92**), lutein (**93**), adenosine (**94**), uridine (**95**), 3-pyridinecarboxamide (**96**), 5-hydroxymethyl-2-furaldehyde (**97**), 5-hydroxymethyl-2-furancarboxylic acid (**98**), *bis*-isothiocyanatomethyl benzene (**99**), and pyropheophorbide-a (**100**). Compound **92** exhibited activity to induce the secretion of insulin. Other compounds isolated from *M. oleifera* were L-tryptophan (**101**), benzyl β-D-glucopyranoside (**102**), benzyl-β-primeveroside (**103**), (+)-pinoresinol-4-O-β-D-glucopyranoside (**104**), isolariciresinol-3a-O-β-D-glucopyranoside (**105**), lariciresinol-9-O-β-D-glucopyranoside (**106**), fluoropyrazine (**107**), (10-hydroxy-1,3-dimethylchrysen-3-yl)-5-hydroxypentan-1-one (**108**), hexademethylated 3β,11β-dihydroxyfriedelane (**109**), 6,7-dipropanone-5-hydroxyphenyl-3-methylphenanthrene-1-carboxylic acid (**110**), (2*R*)-2-phenylmethoxybutane-1,4-diol (**111**), (2*S*)-2-phenylmethoxybutane-1,4-diol (**112**), 2-hexenyl-β-D-glucopyranoside (**113**), omoringone (**114**), 1,3-dibenzyl urea (**115**), 1-hydroxy-3-phenylpropa*n*-2-yl benzoate (**116**), 1-octadecene (**117**), 2,3,4-trihydroxybenzaldehyde (**118**), 3,4-dihydroxy benzonitrile (**119**), 3,7,11,15-tetramethyl-2-hexadece*n*-1-ol (**120**), 3-hydroxy-β-ionone (**121**), *N*-benzyl *S*-ethyl thioformate (**122**), benzyl benzylcarbamate (**123**), methyl-4-hydroxybenzoate (**124**), and pyrrolezanthine (**125**). The structures of these compounds are shown in Figure 11.

Compound **104** displayed a potent inhibition of CYP3A4, with an IC_50_ value of 41.5 µg/mL, while compound **105** exhibited CYP3A4 inhibition with an IC_50_ value of 100 µg/mL. Compound **107** also demonstrated CYP3A4 inhibitory activity, with an IC_50_ value of 72.5 µg/mL. In addition to their enzyme inhibitory effects, compounds **109** and **110** showcased strong antioxidant properties, as indicated by DPPH IC_50_ values of 0.475 and 0.671 mg/mL, respectively. Furthermore, compound **122** exhibited significant antibacterial activity, with MIC values of 32 µg/mL against pathogens such as *S. dysenteriae*, *S. boydii*, and *S. aureus* [13,25,30,37,41,43,51,58,63,64,65,66,67,68,69].

## 3. Pharmacological Properties

### 3.1. Anti-Hemorrhage

Anti-hemorrhage properties in medicine are used to prevent excessive bleeding due to injury or surgery. Excessive bleeding could lead to death. Adeyemi et al. investigated the anti-hemorrhage properties of *M. oleifera* extracts. The experiment was studied by utilizing the venom of *Echis ocellatus*. The result reported that the ethanol extract of *M. oleifera* showed the highest effictiveness in neutralizing hemorrhage, with a dose of 800 mg/kg for 2 mL of 0.22 mg/kg venom. They also investigated the incubation factor and demonstrated its enhanced potency when pre-incubated with venom from *E. ocellatus* [43].

### 3.2. Anti-Allergic

Allergy is a condition where the immune system in the body mistakenly identifies substances as harmful and triggers reactions that affect various parts of the body. The development of anti-allergy medicine has been evolving to improve treatment and understand allergy mechanisms. Rani et al. evaluated the effectiveness of the anti-allergic properties of the leaves, pods, and seeds of *M. oleifera*. The extracts were macerated with ethanol at 80% and yielded nine compounds from the isolation. The study showed that the extracts of *M. oleifera* could inhibit the early and late phases of allergic reactions. In particular, the leaf extracts could better suppress the release of β-hexosaminidase (IC_50_ 7.17 µg/mL), IL-4 (IC_50_ 2.32 µg/mL), and TNF-α (IC_50_ 1.2 µg/mL) compared to ketotifen fumarate as a positive control. *M. oleifera* seed extract could inhibit histamine release better compared to other extracts and the positive control, with an IC_50_ value of 5.97 µg/mL. Further study showed that compared to other isolated compounds, glucomoringin (**28**) had a better inhibition against beta-hexosaminidase in the early phase and TNF-α release in the late phase (IC_50_ 10.43 µg/mL); meanwhile, quercetin (**41**) had a better inhibition against histamine release (IC_50_ 7.77 µg/mL). In the last allergic phase, β-sitosterol-3-*O*-glucoside (**83**) showed a better inhibition against IL-4 release compared to other isolated compounds, with an IC_50_ value of 7.33 µg/mL [50].

### 3.3. Antimicrobial

Antimicrobial properties of compounds always gain interest due to their crucial role in preventing infectious diseases. In an era where microorganisms keep evolving and show resistance to antimicrobial agents, the development of natural antimicrobial agents has become urgent. Various parts of *M. oleifera* extracts with antibacterial activity were studied against *E. coli* and *S. aureus* using the agar well diffusion method. The study showed that the 80% methanol extract of leaves, pulp, and seed had the best inhibition against *E. coli*. The 70 and 80% methanol extract of flowers showed the same value against *S. aureus*, and the aqueous pulp extract showed a better result against *S. aureus* compared to other extracts [70]. Abadallah and Ali studied the comparison of aqueous and ethanol extracts of *M. oleifera* against several bacteria. The MIC value and zone inhibition of the ethanol extract showed better results compared to the aqueous extract. *Shigella* spp. was the most susceptible to both extracts [71]. The extracts of *M. oleifera* showed potential for antibacterial and anti-fungal properties, as shown in Table 2.

The antiviral activities of *M. oleifera* extracts against several viruses are shown in Table 3.

### 3.4. Anthelminthic

Parasitic worm infections can infect various parts of the body, which could lead to health issues for the host. This could lead to the economic loss of livestock. To prevent these losses, the development of anthelmintic medicine has gained interest due to parasitic worm that affect not only affect livestock but also human health. Utilizing earthworms 3–5 cm in length and 0.1–0.2 cm in width, Nilani et al. evaluated the anthelminthic properties of the seed oil *M. oleifera*. The seed oil was divided into two concentrations, 25 and 50 mg/mL, they exhibit anthelminthic properties, with paralysis times of 21 and 16 min respectively while the death time 30 and 24 min, respectively. The study also reported that oleic acid (**57**) at a concentration of 25 mg/mL contained in seed oil showed a paralysis time 23 min and a death time 33 min [36].

### 3.5. Antihypertensive

Hypertension is a cardiovascular disease that causes sustained blood pressure levels. It could lead to health complications, including heart disease, kidney, and stroke. Randriamboajonvy et al. utilizing spontaneous hypertensive rats (SHR) as experiment model to demonstrate the effect of *M. oleifera* seed oil. The result showed a significant reduction in nocturnal heart rate without a change in diurnal heart rate after ten days of treatment. The use of seed oil in SHR increased the capacity of the left ventricle during diastole, which was substantially lower in SHR rats as in comparison with WKY (control) rats. Ejection fraction, a measure of systolic ventricular function, was substantially reduced in both SHR groups (control and seed oil-treated) in comparison with to WKY rats. This suggests that seed oil treatment did not have a positive effect on systolic ventricular function in SHR. The increased isovolumic relaxation time, indicative of diastolic function impairment in SHR, was completely reversed by seed oil treatment. *M. oleifera* seed oil treatment also led to a reduction in cardiomyocyte size in SHR seed oil-treated hearts compared to those in SHR control hearts. Furthermore, the study explored the potential involvement of peroxisome proliferator-activated receptor (PPAR) signaling pathways in seed oil’s protective effect against cardiac fibrosis in SHR. The expression of PPARα and PPARδ in cardiac tissue was assessed, revealing increased staining in the left ventricle of SHR seed oil-treated rats compared to SHR controls. These findings collectively suggest a beneficial impact of *M. oleifera* seed oil on cardiac structure and function in SHR, accompanied by an upregulation of PPAR-α and δ signaling pathways [37].

Acuram et al. studied the antihypertensive properties of methanol and ethyl acetate extracts related to inhibition of angiotensin converting enzyme (ACE), furthermore, blood pressure was also investigated. The hypertension was induced in mice with N^ω^-nitro-L-arginine methyl ester (L-name). The result demonstrated that compared to methanol extract, ethyl acetate showed more significant inhibition of ACE and lowering blood pressure on the last day [85].

### 3.6. Antileishmanial

Infection from the genus Leishmanial affecting health issues in tropical and subtropical regions such as Asia, Africa, America, and Mediterranean. This infection is transmitted from sandflies to humans. Kaur et al. studied the anti-leishmanial properties of *M. oleifera* extract against promastigotes of *Leishmania donavani*. The roots were extracted with 70% ethanol and the leaves were extracted with methanol. The roots ethanolic extract and the leaves’ methanolic extract exhibited moderate inhibitory activity, with IC_50_ values of 83.0 and 47.5 μg/mL, respectively. Upon fractionation, the methanolic extract of leaves showed enhanced antileishmanial activity, particularly in its ethyl acetate fraction, which displayed increased potency with an IC_50_ value of 27.5 mg/mL. Niazin was isolated from ethyl acetate fraction gave antileishmanial properties with IC_50_ value of 5.25 mM [41].

### 3.7. Wound Healing

Tofiq et al. macerated the leaves of *M. oleifera* with 70% ethanol. The experiment was conducted in 7 groups and observed wound healing properties. The result demonstrated that ointment with 10% concentration of extract formulation showed a better effect, representing less scarring, brighter skin, and more regenerated hair follicles compared to gentamicin ointment. Ramadhany et al. made *M. oleifera* leaves extract by soxhlet with ethanol. The extract was made into 4 and 15% gel and investigated their wound healing properties on gingival wounds. The study analyzes the neutrophils, fibroblasts, angiogenesis, and epithelial thickness for seven days. It resulted in 15% *M. oleifera* gel extract having a better effect in reducing neutrophils, increasing the number fibroblasts and angiogenesis. However, 4% *M. oleifera* gel extract was better at increasing epithelial thickness [86].

### 3.8. Antioxidant

Antioxidant properties are used to decrease oxidative stress, which could lead to damaging tissues. Antioxidants function as electron donors to free radicals and neutralize them. Thus, made antioxidant properties become one of most researched topics. Antioxidant activities of *M. oleifera* by several bio-assay methods are shown in Table 4.

### 3.9. Anti-diarrheal

Misra et al. evaluated the anti-diarrheal potential of *M. oleifera*. Leaves of *M. oleifera* were extracted with petroleum ether and then subjected to ethanol for seven days. The animal models were divided into six groups, as control and as doses of extract, and castor oil was used to induce diarrhea. The study showed that the ethanol extract successfully acted as an anti-diarrheal within 52 min, with an extract dose 150 mg/kg, and showed total stools of 0.130 mg [90].

### 3.10. Hepatoprotective

Pari and Kumar conducted an evaluation of the hepatoprotective properties of an ethanol extract obtained from *Moringa oleifera* leaves in rats with liver damage caused by anti-tubercular medications such as isoniazid (INH), rifampicin (RMP), and pyrazinamide (PZA). This extract had considerable protective effects when administered orally, as indicated by its impact on numerous parameters. This included the serum levels of glutamic pyruvic transaminase (alanine aminotransferase), glutamic oxaloacetic transaminase (aspartate aminotransferase), bilirubin, and alkaline phosphatase, as well as the levels of lipids and lipid peroxidation in the liver [91].

Khalid et al. assessed the potential protective effects of *M. oleifera* leaf powder and a 70% ethanol extract of *M. oleifera* leaves in alleviating liver and kidney dysfunction induced by polycystic ovary syndrome (PCOS) in female albino mice. PCOS was induced by administering intramuscular injections of testosterone enanthate at a dose of 1.0 mg/100 g body weight for a duration of thirty-five days. The study conducted assessments of renal function (RFT), liver function (LFT), and the oxidative stress biomarker malondialdehyde (MDA) in the serum at intervals of 0, 7, and 14 days. The mice that received treatments of *M. oleifera* exhibited significant reductions in the levels of alanine aminotransferase (ALT), aspartate aminotransferase (AST), alkaline phosphatase (ALP), total bilirubin, urea, and creatinine compared to the PCOS-induced controls. Conversely, there was a notable increase in the levels of total protein, albumin, globulin, and the albumin/globulin (A/G) ratio. Furthermore, oxidative stress levels exhibited significant reductions in response to treatments, exposure duration, and their combined effect. The findings of this study suggest that both *Moringa oleifera* leaf powder and extract have the potential to reduce oxidative stress and improve renal and hepatic activity in female albino mice with PCOS-induced dysfunction [58].

### 3.11. Anti-Inflammatory

Inflammation is a recognized physiological reaction that serves to safeguard the body against infections and promote the healing of tissue injuries. However, persistent or chronic inflammation can potentially support the growth of various disorders and diseases associated with inflammation. Sulaiman et al. assessed the anti-inflammatory potential of an *M. oleifera* aqueous extract. The assay was conducted by carrageenan-induced paw edema. The extract was made at different doses and showed inhibitory effects in a dose-dependent manner. At dose 100 mg/kg, after five hours, the inhibition showed a value of 50%. The suggested anti-inflammatory mechanism was the extract which contained flavonoids with an inhibitory action against NF-k [64].

Fard et al. studied the anti-inflammatory potential of an *M. oleifera* extract on macrophages stimulated with lipopolysaccharide (LPS) in RAW264.7 macrophages. The anti-inflammatory properties of ethanol extracts derived from *M. oleifera*’s bioactive leaves were assessed by examining their ability to inhibit nitric oxide (NO) production using the Griess reaction and to modulate the expression of pro-inflammatory mediators in macrophages. The ethanol leaf extract exhibited a significant inhibition of various inflammatory markers, including NO production, as well as prostaglandin E2, TNF-α, IL-6, and IL-1β secretion. Concurrently, the bioactive extract dose-dependently stimulated the production of IL-10. Furthermore, the ethanol extract effectively suppressed the protein expression of inflammatory markers such as inducible NO synthase, cyclooxygenase-2, and NF-kB p65, also in a dose-dependent manner [65]. 

Previously, Xu et al. investigated the antioxidant properties of *M. oleifera*, and also investigated its anti-inflammatory properties. The study was conducted by determining the NO production of RAW264.7 macrophages. The study revealed that the leaf extract gave a lower NO production, at 100 µg/mL, while the seed extract presented higher NO production. It concluded that the leaf extract had better anti-inflammatory properties compared to the seed extract [46].

An ethyl acetate fraction of *M. oleifera* was assessed to have anti-inflammatory potential against LPS-induced macrophages. The study showed that macrophages which were treated with the ethyl acetate fraction exhibited a reduction in the production of pro-inflammatory mediators. This reduction was observed between the mRNA and protein levels. The study findings indicated that the fraction downregulated the mRNA expression of various inflammatory markers, including IL-1, IL-6, TNF-α, PTGS2, NF-kB P50, and RelA. Furthermore, the fraction effectively inhibited the expression of inflammatory mediators such as IL-6, TNF-α, and cyclooxygenase-2. Notably, the fraction effects included the inhibition of IkB-α phosphorylation and the ability to decrease the expression of nuclear factor NF-kB p65, thereby impeding its nuclear translocation [52].

### 3.12. Anti-Diabetic

One of the traditional usages of *M. oleifera* was as a diabetic medicine. Gupta et al., and Al-Malki and El Rabey studied the proper effects of *M. oleifera* as an anti-diabetic medicine. Pod and seed powders of *M. oleifera* were used in various doses. The animal model used was a streptozotocin-induced diabetic male rat. The parameters used were the determination of IL-6, immunoglobulin A, immunoglobulin G, fasting blood sugar, glycosylated hemoglobin, albumin, potassium, sodium, creatinine, and uric acid. Both studies showed that *M. oleifera* extracts could restore the abnormalities to a slightly normal level; however, a higher dose was suspected to be more effective. The water consumption of all subject groups also returned to normal after treatment [59,60].

Previously, Hamed et al. examined the antioxidant properties of *M. oleifera* leaf extracts. Furthermore, they also investigated the anti-diabetic activity of purified flavonoids from crude extracts by determining their α-glucosidase inhibitory effects. The results showed that purified flavonoids inhibited α-glucosidase by 54.41% at 100 µg/mL and 99.01% at 800 µg/mL in an uncompetitive manner [48]. Chen et al. reported that the ethanol leaf extracts of *M. oleifera* could be used as anti-diabetic medicine. They investigated the activity by determining the α-glucosidase value. The study showed an IC_50_ value of 123 µg/mL [61].

### 3.13. Anticancer

Jung evaluated the anticancer properties of *M. oleifera* leaf extracts against A549 human lung cancer cells. The evaluation was conducted by MTT assay, and the changes and apoptotic effects were observed. The observation revealed that *M. oleifera* extract treatment resulted in a dose-dependent downregulation of caspase-3 and an upregulation of cleaved caspase-3, indicating an induction of apoptosis. There was a significant dose-dependent downregulation of Akt, p-IkB, NF-kB, p-Erk, β-catenin, and cyclin D1, all of which play roles in cell survival and proliferation. Treatment with soluble *M. oleifera* extracts for 48 h resulted in a significantly reduced release of reactive oxygen species (ROS) in comparison to the untreated control group. This implies a decrease in oxidative stress. In summary, the study concluded that MOL treatment induced apoptosis, inhibited the growth of tumor cells, and reduced the levels of internal reactive oxygen species (ROS) in human lung cancer cells. These outcomes emphasize the potential of *M. oleifera* leaf extracts as a promising candidate for further research and development in the context of lung cancer therapy [66]. The anticancer activities of *M. oleifera* against several cancer cell types are shown in Table 5. 

Based on the data attained by Do et al., as shown in Table 3, this study demonstrated that *M. oleifera* extract induced apoptosis in A375 cells, as evidenced by chromatin condensation and the externalization of phosphatidylserine (PS), which are characteristic features of apoptotic cell death. The apoptosis process was initiated by the activation of caspase-9 and caspase-3/7, the cleavage of PARP, and the translocation of apoptosis-inducing factor into the nucleus. These findings provide insights into the apoptotic mechanisms triggered by *M. oleifera* extract in A375 cells [69]. Meanwhile, Pappas et al. assessed the gene expression of *M. oleifera* aqueous wild and cultivated leaf extracts against human pancreas cancer cells AsPC-1, MCF-7, and HCT-116 colon cancer cells. The aqueous extract exerted its effects by down-regulating p53 expression in all tested cell lines and by dow*n*-regulating c-myc in AsPC-1 cells. Additionally, specific marker genes associated with each cell line, such as BRCA-1 in MCF-7, mta-1 in AsPC-1, and Ki-67 in HTC116 cells, were dow*n*-regulated. Furthermore, the survivin (BIRC5) gene, an inhibitor of apoptosis, was dow*n*-regulated in all three cell lines, suggesting a shared target mechanism of Moringa constituents across these cell lines [92].

Kumar et al. discovered that an extract of *M. oleifera* leaves demonstrated effective inhibition of Dalton lymphoma (DL) cell proliferation. This inhibition was characterized by alterations in mitochondrial membrane potential (ΔΨm) and noticeable changes in overall cell morphology. Notably, DL cells treated with the extract experienced cell cycle arrest at the G2/M phase and a significant upregulation in the expression of p53 and p21. Furthermore, the treatment resulted in increased levels of pro-apoptotic markers, including Bax, Cytochrome-c (Cyt-c), and caspase-3, while reducing the expression levels of the anti-apoptotic Bcl-2 protein. These changes strongly suggest the induction of apoptosis in DL cells. Mechanistically, the anticancer efficacy of *M. oleifera* extract was attributed to the inactivation of the MEK/ERK-mediated pathway in DL cells. Additionally, it is noteworthy that the inhibition of DL growth by MOML was accompanied by apoptosis induction and improvements in hematological parameters in DL-induced mice [99].

## 4. Utilization of *M. oleifera* in Dental Health

Research on several bacteria that are pathogenic to dental health has been carried out, such as *Streptococcus mutans*, *Enterococcus faecalis*, *Staphylacoccus aureus*, and others. Table 6 shows some data on the antibacterial activity of *M. oleifera* against oral pathogenic bacteria.

Jwa evaluated the aqueous and ethanol extracts of *M. oleifera* against *S. mutans,* which were found in the cariogenic biofilm of dental caries. Both extracts reduced the bacteria’s growth at concentrations of 25 and 6.25 µg/mL, respectively. At the same concentration, heated ethanol extract exhibited inhibitory activity better than non-heated extracts. This study showed that the ethanol extract was more effective than the aqueous extract against *S. mutans* [109]. Soraya et al. investigated the anti-bacterial properties of *M. oleifera* gel in inhibiting the growth of *S. mutans,* which were involved in the pathogenesis of dental caries. After 48 h, it was observed that the 12.5% concentration exhibited the highest effectiveness in reducing *S. mutans* growth. Within 24 h, the 6.25 and 3.125% concentrations displayed remarkable capabilities in suppressing *S. mutans* growth. Notably, the 6.25% concentration showed superior efficacy in reducing the formation of *S. mutans* biofilms. The application of *M. oleifera* gel extract created conditions in which *S. mutans*, a commensal bacterium, struggled to form a biofilm, with inhibition levels surpassing 70%. This was evidenced by the absence of substantial biofilm development. It is worth mentioning that at all tested concentrations, *M. oleifera* exhibited a toxic effect on *S. mutans* cells. The ethanol extract gel of *M. oleifera* demonstrated the ability to curtail both the growth and biofilm formation of *S. mutans* on tooth surfaces while concurrently exerting toxicity on *S. mutans* cells, potentially due to the presence of anti-bacterial compounds [110]. The anti-bacterial properties of herbal toothpaste, formulated with *M. oleifera* root essential oil, were assessed against bacteria commonly associated with tooth plaque, namely *S. mutans* and *S. aureus*. The Muller Hinton agar well diffusion method was used for this evaluation The findings of this study demonstrated that *S. mutans* exhibited susceptibility to the herbal toothpaste, as indicated by a significant inhibition zone measuring 31 mm [111].

Alharbi et al. examined the antimicrobial efficacy of *M. oleifera* leaf extract, octenidine dihydrochloride (OCT), sodium hypochlorite (NaOCl), and their combinations as an intracanal irrigant against *E. faecalis*. Decoronation and root canal preparation were performed on single-rooted mandibular premolars. After autoclaving, each root specimen was inoculated with *E. faecalis* and incubated at 37 °C for 48 h. Subsequently, based on the irrigation solution used, the specimens were split into six groups: 2.5% NaOCl (Group 1), 0.1% OCT (Group 2), *M. oleifera* leaf extract (Group 3), a combination of *M. oleifera* extract and 1.25% NaOCl (Group 4), a combination of *M. oleifera* extract and OCT (Group 5), and normal saline (Group 6). Both *M. oleifera* extract and 0.1% OCT demonstrated antibacterial effects against *E. faecalis* comparable to 2.5% NaOCl and could be considered as potential root canal irrigants. Furthermore, combination groups exhibited superior anti-microbial activity compared to individual irrigants [112].

Rochyani investigated the inhibitory potential of *M. oleifera* leaf extract on the biofilm formation of *E. faecalis* bacteria. The experiment was divided into several groups, including a negative control group (CMC solvent 0.1%), a positive control group (ChKM), and four test groups extracts of 20, 40, 60, and 80%, respectively. The results indicate that the *M. oleifera* leaf extract demonstrates a significant inhibitory effect on the biofilm formation of *E. faecalis* bacteria. Notably, the inhibitory effect observed in the 80% extract was found to be substantially greater than that achieved by the positive control group using ChKM [113].

Shanmugapriya et al. investigated the antimicrobial effects of *M. oleifera* extracts on pooled plaque collected from orthodontic patients. To achieve this, *M. oleifera* extracts were prepared through maceration. Subgingival plaque samples were collected, and the microorganisms present were cultured under anaerobic conditions. The microorganisms were subsequently subjected to treatment with the extracts, and their MIC and MBC were determined. Additionally, the cytotoxic effects of the extracts were assessed using a brine shrimp assay. The results of the study revealed that the 5% aqueous extract of *M. oleifera* exhibited a dose-dependent antimicrobial activity against oral anaerobic organisms. Notably, this anti-microbial effect became more pronounced with longer exposure times of the treated samples. Furthermore, in the cytotoxicity assay, the aqueous extract showed superior performance in lower concentrations in comparison to the ethanol extract. This was evident from the higher number of live nauplii observed in the aqueous extract group, indicating its lower cytotoxicity [114].

Sopandani et al. evaluated the antibacterial efficacy of *M. oleifera* extract at various concentrations (25, 50, 75, and 100%) when used as an irrigation solution against *E. faecalis* within the root canal ex vivo. Quantitative polymerase chain reaction (qPCR) was employed to assess the population of *E. faecalis* within the root canal following treatment with *M. oleifera* extract. The results indicated that *M. oleifera* extract solutions at concentrations of 75 and 100% exhibited comparable effectiveness to a 5.25% sodium hypochlorite (NaOCl) solution as a positive control [114].

Rieuwpassa et al. assessed the effectiveness of *M. oleifera* leaf extract in modulating the anti-inflammatory cytokine IL-1 of the bacteria *Porphyromonas gingivalis*, a key contributor to chronic periodontitis. The study revealed a significant variation in IL-1 levels across different observation days. Administration of the extract led to a reduction in pro-inflammatory cytokine IL-1 levels, as evident from the observations on days D0, D1, D3, D5, and D7 in the experimental Wistar rats induced with *Porphyromonas gingivalis* bacteria [115].

Kumar et al. evaluated the antibacterial effectiveness of a 5% *M. oleifera* mouthwash enhanced with silver nanoparticles against oral aerobic microorganisms. The mouthwash was prepared by utilizing a 5% *M. oleifera* aqueous extract for the synthesis of silver nanoparticles. The characterization of the mouthwash was performed through scanning electron microscopy analysis and energy dispersive X-ray analysis. To assess its anti-bacterial properties, the mouthwash was tested against *S. mutans*, *S. aureus*, *E. faecalis*, and *C. albicans* using the agar well diffusion assay. The results indicated that the 5% *Moringa oleifera*-silver nanoparticle mouthwash exhibited a pronounced effect on *S. aureus* and a comparable impact on *S. mutans* [114].

## 5. Conclusions

*M. oleifera* has a variety of pharmacological effects, such as anti-hemorrhage, anti-allergic, antimicrobial, anthelmintic, antihypertensive, antileukemia, antioxidant, anti-diabetic, hepatoprotection, anti-inflammatory, and anticancer effects. This plant is effective against dental infections. Antimicrobial research against microbes that cause dental infections has been carried out on the leaves of *M. oleifera* both in aqueous and ethanol extracts. This plant is reported to be active in vitro to inhibit several oral bacteria such as *E. faecalis*, *S. mutans*, *P. gingivalis*, *S. aureus,* and *C. albicans*, and has been tested ex vivo. The chemical components contained in *M. oleifera* are phenolics, glucosinolates, flavonoids, fatty acids, esters, alkaloids, sterols, terpenes, and several other compounds. Based on the pharmacological effects and chemical components contained in *M. oleifera*, this plant has the potential to be further developed to produce an antibacterial agent product, especially for dental health. The search for bioactive compounds in *M. oleifera* that have a major role as antibacterials in oral pathogens can be carried out through isolation. Furthermore, both extracts and isolated compounds from *M. oleifera* can be further traced for their activity through in vitro, in vivo, and clinical trials.

## Figures and Tables

**Table 1:** Traditional usage of *M. oleifera*.

Part of Plant	Usage	References
Seed	Skincare, haircare, fertilizer, cure for eye disease, fever, snake bite, headache, bladder, ulcer, gastritis, gout, stimulant, antispasmodic, stomachache, anemia, joint pain, hypertension, water purification.	[2,3,4]
Leaf	Wound healing, snake bites, stimulation, breast milk production, diarrhea, animal feed, constipation, bronchitis, glandular swelling, rheumatism, influenza, food, malaria, arthritis.	[2,3,4,5,6]
Root	Anticoagulation, wound healing, laxative, diuretic, toothache, cold, sores, asthma, bronchitis, epilepsy, urinary discharge, laxative, antiparalytic, cardiac tonic.	[3,4,5]
Pod (Fruit)	Diabetic, antipyretic, asthma, spleen, skin tumor, joint pain.	[4,5]
Flower	Stimulant, tonic, cholagogue, cold, inflammation, muscle disease, tumor, cholera.	[4,5]
Bark (Stem)	Heart compilation, fever, eye disease, digestive disorder, animal feed, headache, hypoglycemia, toothache.	[4,5,6]

**Table 2:** Antimicrobial activity of *M. oleifera* against (A) Gram negative bacteria, (B) Gram positive bacteria, and (C) fungi.

*M. oleifera*	Microorganism	Inhibition Zone (mm)	MIC (mg/mL)	MBC (mg/mL)	References
**(A) Gram Negative Bacteria**					
Stem methanol extract	*Vibrio cholerae* *Vibrio mimicus*	- -	2.50 1.25	- -	[72]
Leaves ethanol extract	*V. cholerae* *V. mimicus*	- -	0.08 5	- -	[72]
Pods ethanol extract	*V. cholerae* *V. mimicus*	- -	0.31 2.5	- -	[72]
Flower chloroform extract	*V. cholerae* *V. mimicus*	- -	0.63 1.25	- -	[72]
Ethanol extract	*Proteus mirabilis* *Fusarium* sp.	- 12	3.75 μg/mL -	- -	[55]
Methanol extract	*Burkholderia cepacian* *Yersinia enterocolitica*	19 19	- -	- -	[55]
Aqueous extract	*Proteus vulgaris*	15	-	-	[55]
	*Escherichia coli*	15	-	-	
	*Yersinia enterocolitica*	15	-	-	
	*Serratia rubidaea*	15	-	-	
	*Salmonella pollum*	15	-	-	
	*Pullarum* sp.	5	-	-	
	*P. mirabilis*	-	3.75 μg/mL	-	
Root powder extract	*E. coli*	-	87%	-	[73]
Nanoparticles loaded to extract	*Aspergillus niger*	55	-	-	[74]
Ethanol extract	*Aeromonas cavie* *Vibrio parahaelomyticus*	23.8 21.9	- -	- -	[75]
Aqueous extract	*Aeromonas cavie* *V. parahaelomyticus*	22.3 20.7	- -	- -	[75]
Methanol pod extract	*P. aeruginosa*	22	-	-	[76]
Acetone extract	*E. coli* *Enterobacter cloacae* *P. vulgaris*	- - -	5 5 5	5 5 5	[77]
Leaf ethanol extract	*E. coli*	18.3	-	-	[78]
Leaf methanol extract	*E. coli*	19	-	-	[78]
Leaf aqueous extract	*E. coli*	14	-	-	[78]
Aqueous extract Ethanol extract	*E. coli*	18.25 27.75	25 390 μg/mL	- -	[79]
Aqueous extract Ethanol extract	*Klebsiella pneumoniae*	21.75 28.5	50 780 μg/mL	- -	[79]
Aqueous extract Ethanol extract	*Citrobacter* sp.	20.65 19.5	50 390 μg/mL	- -	[79]
Aqueous extract Ethanol extract	*P. vulgaris*	14.75 24.75	25 780 μg/mL	- -	[79]
Aqueous extract Ethanol extract	*P. aeruginosa*	17.5 22.25	25 780 μg/mL	- -	[79]
Aqueous extract Methanol extract	*Staphylococcus aureus*	20 24	- -	- -	[79]
Aqueous extract Methanol extract	*E. coli*	18 16	- -	- -	[80]
Methanol extract	*Klebsiella* spp.	25	-	-	[81]
Ethanol extract	*Enterococcus faecalis*	27.5	10% (*w*/*v*)	-	[82]
Ethanol extract	*Salmonella typhi*	8	8	8.5	[83]
Chloroform extract	*Shigella dysenteriae*	-	1500 μg/mL	2000 μg/mL	[31]
Ethanol seed extract	*E. coli* *Shigella flexneri*	16 15	100 100	- -	[25]
**(B) Gram Positive Bacteria**					
Methanol extract	*Aspergillus flavus*	12	-	-	[55]
Nanoparticles loaded to extract	*A. flavus*	55	-	-	[74]
Ethanol extract	*S. aureus* *Enterococcus aureus*	23.3 19.4	- -	- -	[75]
Aqueous extract	*S. aureus* *E. aureus*	25.4 17.8	- -	- -	[75]
Acetone extract	*S. aureus* *Micrococcus kristinae*	- -	5 0.5	5 1	[77]
Leaf ethanol extract	*Bacillus subtilis* *S. aureus*	19 21.3	- -	- -	[78]
Leaf methanol extract	*B. subtilis* *S. aureus*	22 23.6	- -	- -	[78]
Leaf aqueous extract	*B. subtilis* *S. aureus*	12 18	- -	- -	[78]
Aqueous extract Ethanol extract	*Corynebacterium pseudotuberculosis*	22.5 25.65	25 390 μg/mL	- -	[79]
Aqueous extract Ethanol extract	*Corynebacterium ulcerans*	25.5 30.5	25 390 μg/mL	- -	[79]
Aqueous extract Ethanol extract	*S. aureus*	14.75 26.75	50 390 μg/mL	- -	[79]
Aqueous extract Methanol extract	*B. subtilis*	23 23	- -	- -	[80]
Ethanol extract	*Staphylococcus epidermidis*	12	-	-	[80]
Ethanol extract	*S. aureus*	19.5	10% (*w*/*v*)		[82]
**(C) Fungi**					
Aqueous extract	*Candica albicans*	5	-	-	[55]
Ethanol extract	*C. albicans*	-	718.33 μg/mL	-	[74]
Nanoparticles loaded to extract	*C.albicans*	75	-	-	[74]
Methanol pod extract	*Colletotrichum* sp.	14	-	-	[76]
Ethanol extract Aqueous extract	*C.albicans*	1.87 cm 1.87 cm	- -	- -	[63]
Ethyl acetate extract	*Microsporum gypseum* *Rhizopus stolonifer*	9.67 8.67	1.56 6.25	- -	[26]
Methanol extract	*R. stolonifer*	9.66	1.56	-	[26]

Note: - (not tested).

**Table 3:** Antiviral activity of *M. oleifera* against several viruses.

*M. oleifera*	Viral	IC_50_ (μg/mL)	CC_50_ (μg/mL)	EC_50_ (μg/mL)	References
Leaf extract	SARS-CoV-2 “NRC-03-nhCoV”	52.79	111.54	-	[27]
Crude ethanol extract	SARS-CoV-2	12.29	7277	-	[28]
Seed extract	IAVs	-	-	1.27	[29]
Seed extract	H1N1	0.26	-	-	[84]
Aqueous extract	HSV-1 HSV-2	43.2% 21.4%	- -	- -	[30]
Leaf ethanol extract	H9	-	100	-	[33]
Methanol extract	HSV-1F VU-09	- -	724.5 -	74.8 79.6	[34]
Aqueous leaf extract	AqMOL ACV	- -	697.8 >30	721.8 0.48	[35]

Note: - (not tested).

**Table 4:** Antioxidant activity of *M. oleifera* by several bio-assay methods.

*M. oleifera*	Bio-Assay	IC_50_	EC_50_ (mg/mL)	References
Roots extract Leaf extract Stem bark extract	Xanthine oxidase	16 μL 30 μL 38 μL	- - -	[44]
Roots extract Leaf extract Stem bark extract	2-deoxyguanosine	40 μL 58 μL 72 μL	- - -	[44]
*n*-Butanol extract Ethyl acetate extract Petroleum ether extract Aqueous extract	DPPH	92.62% 90.27% - -	0.07 0.08 0.35 0.44	[45]
*n*-Butanol extract Ethyl acetate extract Crude extract	ABTS	99.46% 97.49% 77.82%	0.01 0.04 -	[45]
Petroleum ether extract Aqueous extract	ABTS	- -	0.18 0.29	[45]
*n*-Butanol extract Ethyl acetate extract	Hydroxy radical-scavenging	94.46% 80.68%	- -	[45]
Leaf extract	DPPH	1.87 mg/mL	-	[46]
Leaf extract	FRAP	0.99 mM Fe^2+^/g	-	[46]
Root extract	ABTS	1.24 mg/mL	-	[46]
Ethyl acetate extract Acetone extract	DPPH	526.7 μMol 435.7 μMol	- -	[87]
Ethanol extract	DPPH	0.44 mg/mL	-	[88]
Ethanol extract	Hydroxy peroxide free radical scavenging	0.54 mg/mL	-	[88]
Ethanol extract	FRAP	0.25 mg/mL	-	[88]
Ethyl acetate extract	DPPH	71.9 μg/mL	-	[89]
Ethyl acetate extract	ABTS	54.79 μg/mL	-	[89]

Note: - (not tested).

**Table 5:** Anticancer activity of *M. oleifera* against several cancer cell types.

*M. oleifera*	Bio-Assay	IC_50_ (μg/mL)	Other Values	References
Seed essential oil	HeLa HepG2 MCF-7 CACO-2 L929	442.8 751.9 226.1 1000 1000	23.9% 34.93% 40.48% 50.28% 42.99%	[67,68]
Aqueous extract	HeLa	70	70 μg/mL	[67,68]
Aqueous extract	A375	-	36.40%	[69]
Aqueous extract	Bcl-2	-	0.68 to 0.53-fold	[69]
Aqueous extract	Bax	- -	2.62-fold increase at the m-RNA level; 1.85-fold increase at the protein level	[69]
Aqueous extract	MOE activation of Caspase-3/7	-	Increase 1.75-fold	[69]
Aqueous extract	MOE activation of Caspase-9	-	Increase 1.42-fold	[69]
Aqueous extract	MCF-7 HTC116 AsPC-1	100 125 240	- - -	[92]
Leaf extract	Urethane-induced lung cancer in rats	-	Induced in glutathione 3.8 mg/g, superoxide dismutase 900.6 U/g, and malondialdehyde 172 nmol/g	[93]
Leaf extract	Urethane-induced lung cancer in rats	-	Increase 50% EGFR-mRNA, 10.8% improvements of mucin level and the presence of PCNA-positive cells in lung	[93]
CO_2_ root extract	MCF-7	-	Spanning 100 to 500 μg/mL	[94]
*n*-Hexane extract Chloroform extract Ethyl acetate extract Methanol extract	Hep-2	180.6 190.2 40.2 170.1	- - - -	[95]
Crude extract	HCT116	9.5 (24 h) 5.04 (48 h)	-	[96]
Crude extract	CYP3A4	52.50	-	[54]
Chloroform extract	MCF-7	6.25		
Dicholoromethane extract	MCF-7	5	1.87-fold increase in p53 expression, 1.47-fold increase in Bax expression 1.05-fold increase in cytochrome C levels, 2.21-fold increase in caspase 8 expression	[97]
Ethyl acetate extract	MDA-MB-231	233.5	Increase 44.2% of late apoptotic cells; increased level of cleaved caspase 3 protein, Bax mRNA, and p53 mRNA; decreased anti-apoptotic Bcl-2 protein	[98]

Note: - (not tested).

**Table 6:** Antibacterial activity of *M. oleifera* to some oral pathogenic bacteria.

*M. oleifera*	Microorganism	Inhibition Zone (mm)	MIC (μg/mL)	MBC	References
Ethanol extract	*P. aeruginosa*	21.21	458	-	[100]
Ethanol extract	*S. aureus*	20.55	>1	-	[101]
Aqueous extract	*S. aureus*	12	58.75 mg/mL	-	[100]
Methanol extract	*E. faecalis*	44.83	-	-	[102]
Leaves ethanolic extract	*S. aureus*	19.25	-	-	[103]
	*S. mutans*	13			
Roots ethanolic extract	*S. aureus*	9.25	-	-	[103]
	*S. mutans*	10.50			
Seed ethanolic extract	*S. aureus*	3.25	-	-	[103]
	*S. mutans*	4.75			
Bark ethyl acetate extract	*S. aureus*	16.33	-	-	[104]
Root bark methanolic extract	*S. aureus*	19	12.5 mg/mL	-	[105]
Leaf extract	*S. mutans* biofilm	0.20/(OD 520 nm)	-	-	[106]
Diethyl ether, *n*-Hexane, and Ethyl acetate extract	*S. aureus*	-	15.6	-	[107]
Diethyl ether extract	*E. faecalis*	-	15.6	-	[107]
extract	*S. aureus*	-	15.6	-	[107]
Leaf extract	*E. faecalis*	11.89 (at 100 μg/mL)	-	-	[108]
Leaf extract	*E. faecalis*	35.5 (at 24 h) 48.83 (at 48 h)	75	-	[102]

Note: - (not tested).

## Data Availability

Not applicable.
